# Strain-level *Staphylococcus* differentiation by CeO_2_-metal oxide laser ionization mass spectrometry fatty acid profiling

**DOI:** 10.1186/s12866-016-0658-y

**Published:** 2016-04-23

**Authors:** Nicholas R. Saichek, Christopher R. Cox, Seungki Kim, Peter B. Harrington, Nicholas R. Stambach, Kent J. Voorhees

**Affiliations:** Department of Chemistry, Colorado School of Mines, Golden, CO 80401 USA; Korea Institute of Science and Technology, Hwarangno 14-gil 5, Seongbuk-gu, Seoul 136-791 Republic of Korea; Center for Intelligent Chemical Instrumentation, Department of Chemistry, Clippinger Laboratories, Ohio University, Athens, OH 45701 USA

**Keywords:** MALDI–MS, MOLI MS, *Staphylococcus*, MRSA, Fatty acids

## Abstract

**Background:**

The *Staphylococcus* genus is composed of 44 species, with *S. aureus* being the most pathogenic. Isolates of *S. aureus* are generally susceptible to β-lactam antibiotics, but extensive use of this class of drugs has led to increasing emergence of resistant strains. Increased occurrence of coagulase-negative staphylococci as well as *S. aureus* infections*,* some with resistance to multiple classes of antibiotics, has driven the necessity for innovative options for treatment and infection control. Despite these increasing needs, current methods still only possess species-level capabilities and require secondary testing to determine antibiotic resistance. This study describes the use of metal oxide laser ionization mass spectrometry fatty acid (FA) profiling as a rapid, simultaneous *Staphylococcus* identification and antibiotic resistance determination method.

**Results:**

Principal component analysis was used to classify 50 *Staphyloccocus* isolates. Leave-one-spectrum-out cross-validation indicated 100 % correct assignment at the species and strain level. Fuzzy rule building expert system classification and self-optimizing partial least squares discriminant analysis, with more rigorous evaluations, also consistently achieved greater than 94 and 84 % accuracy, respectively. Preliminary analysis differentiating MRSA from MSSA demonstrated the feasibility of simultaneous determination of strain identification and antibiotic resistance.

**Conclusion:**

The utility of CeO_2_-MOLI MS FA profiling coupled with multivariate statistical analysis for performing strain-level differentiation of various *Staphylococcus* species proved to be a fast and reliable tool for identification. The simultaneous strain-level detection and antibiotic resistance determination achieved with this method should greatly improve outcomes and reduce clinical costs for therapeutic management and infection control.

## Background

Staphylococci are Gram-positive facultative anaerobes comprising 44 species commonly found in soil or on the skin of animals [1]. *S. aureus* is the most pathogenic of the genus and is commonly associated with septicemia, osteomyelitis, endocarditis, and skin infection [2]. Isolates of *S. aureus* are generally susceptible to β-lactam antibiotics, but extensive use of this class of drugs has led to the emergence of resistant strains [3]. In 2011 the Centers for Disease Control and Prevention (CDC) reported 80,461 methicillin-resistant *S. aureus* (MRSA) infections in the U.S. leading to 11,285 deaths. While improved infection control policies decreased clinical MRSA infections by 52 % between 2005 and 2011, there remains a need to rapidly screen patients for *S. aureus* and determine antibiotic resistance.

Culture-, biochemical-, and molecular-based methods are the current standard for clinical MRSA detection. Culture methods offer high specificity, but relatively lengthy turnaround times (TAT) of 24–72 h and the requirement for secondary resistance testing contribute significantly to delays in onset of treatment. A retrospective cohort study of bloodstream infections found that mortality rates rose 7.6 % per hour for every hour of delay in the initiation of effective antimicrobial therapy [4]. Chromogenic agars have been used to slightly decrease TAT to 18–24 h, while also improving specificity, but secondary resistance testing is still required [5].

Some of the most common approaches for analysis of the specific biochemical characteristics of staphylococci include: coagulase and phosphatase activity, hemolysis, nitrate reduction, and aerobic acid production from carbohydrate metabolism [6]. Kloos and coworkers reported a simplified scheme for analyzing the extensive data produced by biochemical results to characterize staphylococci. The commercially available BioMérieux API STAPH-IDENT and American Hospital Supply Corporation MicroScan Systems are based on this approach. The API Staph-IDENT utilizes a battery of 10 microscale biochemical tests, whereas the MicroScan System consists of 27 tests [7]*.* These systems were reported to have accuracies of 88 and 86.4 %, respectively, but also showed inherent limitations [8–10].

In order to improve the specificity and selectivity of *Staphylococcus* detection, molecular methods for analyzing specific genetic markers have been explored. In an attempt to identify *S. aureus* and assay for methicillin resistance, multiplexed PCR has been used to simultaneously target the staphylococcal *nuc* gene, encoding a thermostable nuclease (TNase), and the *mecA* gene, encoding a penicillin binding protein [11]. PCR results agreed with coagulase production and agar screening tests for single-step identification of MRSA. In an attempt to identify coagulase-negative staphylococcal strains (CoNS), one study targeted a 429-bp amplicon of the *sodA* gene encoding the manganese-dependent superoxide dismutase [12]. Clinical isolates and ATCC reference strains were identified with 83 % accuracy in about 8 h. While culturing and biochemical assays offer comparable specificity to results obtained by *hsp60* [13] and 16S rRNA sequencing [14]; TAT is still typically greater than 24 h.

Turnaround time was significantly reduced using phage amplification-based lateral flow immunochromatography (LFI) [15]. This work led to the FDA-approved MicroPhage KeyPath MRSA/MSSA blood culture test [16]. Exploitation of *S. aureus*-specific phage amplification targeting clinical blood isolates allowed for simultaneous identification and methicillin resistance determination with a TAT of 5 h and 98.3 % accuracy [15].

Published reports suggest the rise of non-*S. aureus* infections in clinical studies, some with resistance to multiple classes of antibiotics [17–19]. CoNS are among the most commonly reported bloodstream isolates (37.3 % compared to 12.6 % for *S. aureus*) [20]. These reports place emphasis on the importance of *S. epidermidis, S. saprophyticus*, *S. lugdunensis*, and *S. schleiferi* infection and further demonstrate the need for more rapid techniques for simultaneous species-level *Staphylococcus* identification and antibiotic resistance determination. Bacterial protein-profiling by matrix assisted laser desorption/ionization-time of flight mass spectrometry (MALDI-TOF MS) has been used to identify *S. aureus* and CoNS in prosthetic joint infections [21]. Although this method was relatively rapid, only 52 % highly probable species-level identification was obtained.

A report by Dubois and coworkers using the Bruker Biotyper MALDI-TOF MS protein analysis of 152 staphylococcal isolates correctly identify 151 samples at the species level. These results confirmed their earlier findings using a PCR-based *sodA* gene array [22]. Rajakurna et al. correctly identified a different set of *Staphylococcus* isolates with 97 % accuracy using the MicrobeLynx macromolecule profiling database, developed by Waters Corporation [23].

A MALDI mass spectral-bacterial profiling approach using fatty acids as diagnostic biomarkers rather than proteins was recently reported [24–26]. Employing MALDI with CeO_2_ (metal oxide laser ionization [MOLI] MS) as an in situ saponification catalyst and matrix replacement, bacterial samples were identified to the species level with 97 % accuracy [27]. In a follow up study, suites of *Enterobacteriaceae, Listeria,* and *Acinetobacter* were analyzed in parallel by MOLI MS fatty acid profiling and the Bruker Biotyper protein profiling [28]. The results from this study clearly established fatty acid MOLI MS profiling for strain-level differentiation of closely-related phylotypes with 98–100 % accuracy. In comparison, protein profiling of the same samples correctly identified *Enterobacteriaceae* with 30 %, *Listeria* with 64 % and *Acinetobacter* with 66 % accuracy at the species level.

The present study describes MOLI MS CeO_2_ fatty acid profiling of 31 non*-aureus Staphylococcus* strains and 19 *S. aureus* strains (nine MRSA and ten MSSA). A fuzzy rule building expert system (FuRES) [29] and a self-optimizing partial least squares discriminant analysis (PLS-DA) [30] were used for classification.

## Results and discussion

### Spectral analysis

**Fig. 1:**
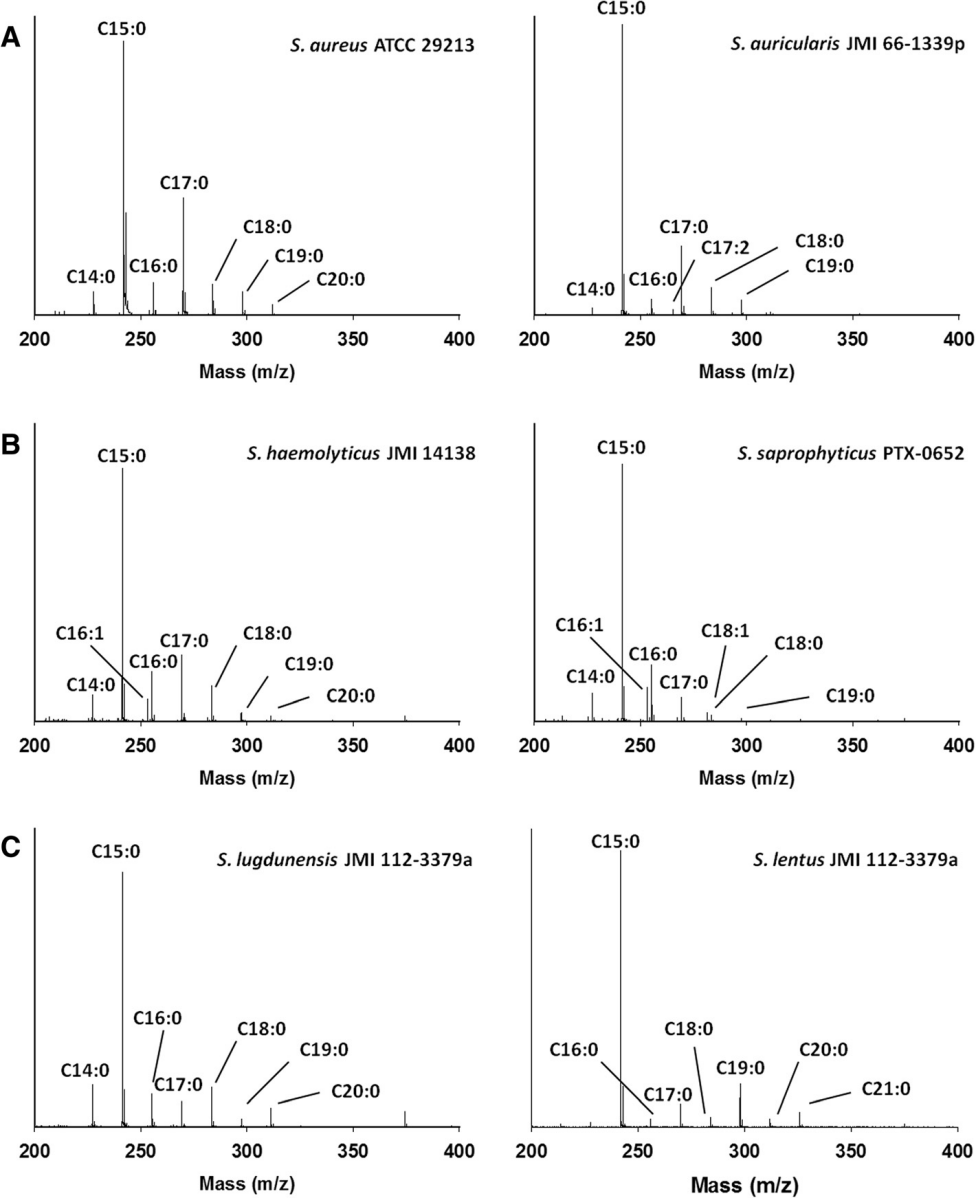
Representative fatty acid profiles. Fatty acids are labeled with respect to chain length and degree of unsaturation. (**a**) Group 1, (**b**) Group 2, (**c**) Group 3

MOLI MS was used to analyze 14 *Staphylococcus* extracts listed in Table 1A to develop FA profiles. For the 14 *Staphylococcus* species, the spectra (data not shown) contained similar fatty acids. C15:0 was common to all spectra as the base peak, while the other FAs, listed in Table 2, ranged from 0 to 30 % relative abundance. The intensities of FA peak distribution allowed the spectra to be visually divided into three distinct categories: Group 1: *S. aureus*, *S. auricularis, S. capitis, S. epidermidis,* and *S. shleiferi*, which were all observed to have similar respective C16:0, C17:0 and C18:0 ratios; Group 2: *S. harmolyticus, S. haemolyticus*, *S. hyicus,* and *S. saprophyticus*, which displayed the highest prevalence of unsaturation consisting of 10-38 % unsaturated FAs; and Group 3: *S. lugdunensis, S. lentus, S. simulans*, and *S. warneri*, which each exhibited a unique defining characteristic absent from the other two groups. Figure 1 shows two representative spectra for each of the three groups. As visual examples, slight differences in the relative abundance of minor FAs for Group 1 enhanced differentiation. Figure 1a illustrates differentiation of *S. aureus* and *S. auricularis* by the appearance of C17:2 and C20:1 in the latter. As shown in Fig. 1b, minor FAs were crucial in separating Group 2 organisms. For example, *S. haemolyticus* was differentiated from *S. saprophyticus* by the absence of C20:0 as well as a decrease in C18:0 and increase in C18:1 in the latter. Figure 1c illustrates the differentiation of Group 3 organisms. *S. lugdunensis,* was distinguished from *S. lentus* by C14:0, which was the second most abundant FA with respect to C15:0, encompassing 20 % of the relative abundance, as well as by the appearance of C21:0 in *S. lentus*. Visual analysis of the respective ratios of FAs provided a qualitative basis for bivariate analysis, but multivariate statistics were needed to process complex data sets.

### Species-level differentiation

**Fig. 2:**
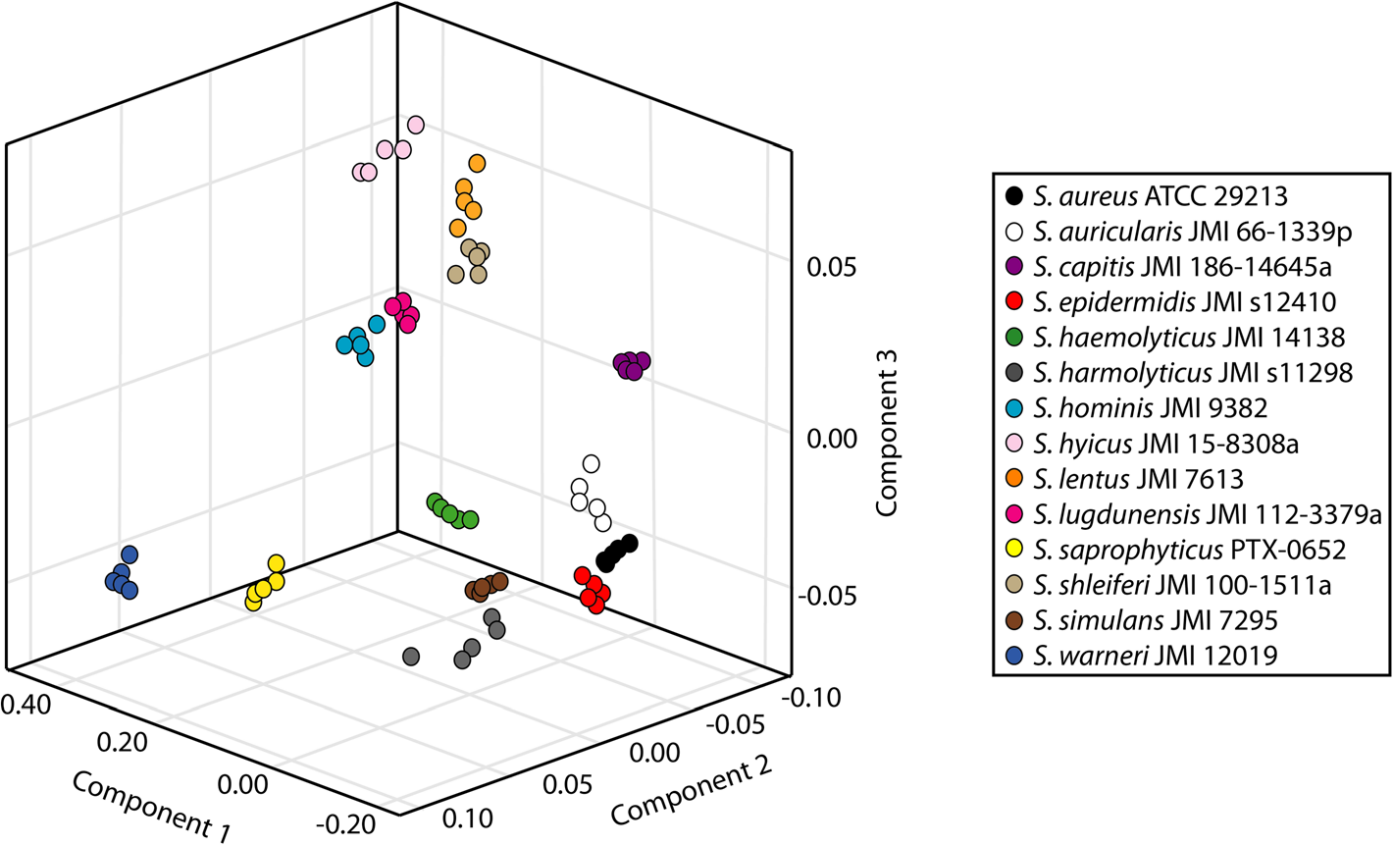
Species-level PCA differentiation of 14 *Staphylococcus* isolates

**Fig. 3:**
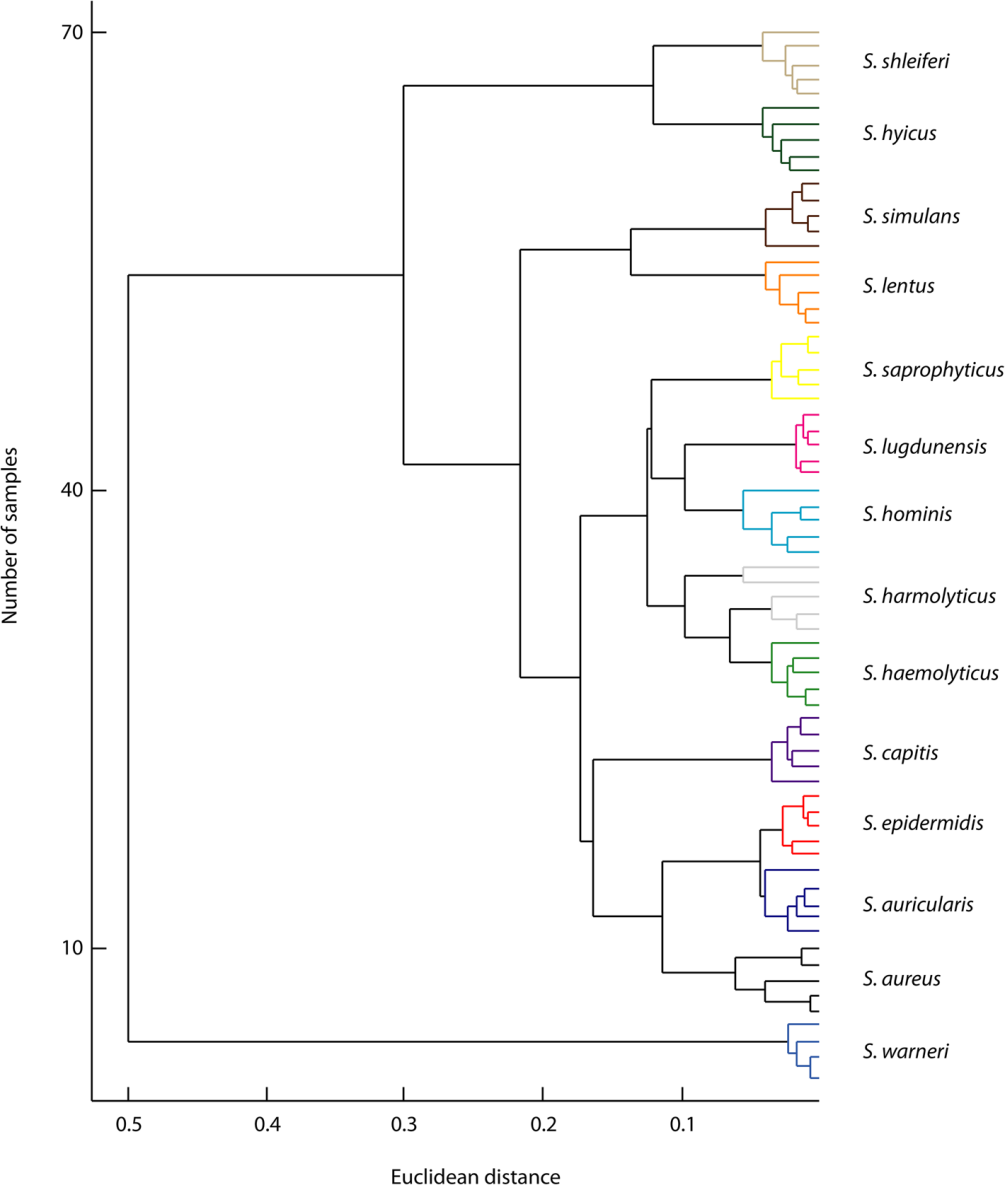
Dendrogramatic representation of *Staphylococcus* species differentiation. Branch lengths were determined using average linkages and Euclidean distance

Principal component analysis (PCA) was employed to classify *Staphylococcus* at the species-level. A score plot of the first three components, which encompassed 93.6 % of total variance, is shown in Fig. 2. Colored points represent individual replicates of each bacterial species. The degree of separation was indicated by the distinct clustering of members of the same species (inner variance) and the distance between different species (outer variance). All species clearly plotted in unique space, which was supported by the 100 % classification rate obtained by LOSOCV. Figure 3 shows a dendrogram based on Euclidean distances between spectra, which demonstrated classification of the profiles into well-defined clusters.

**Fig. 4:**
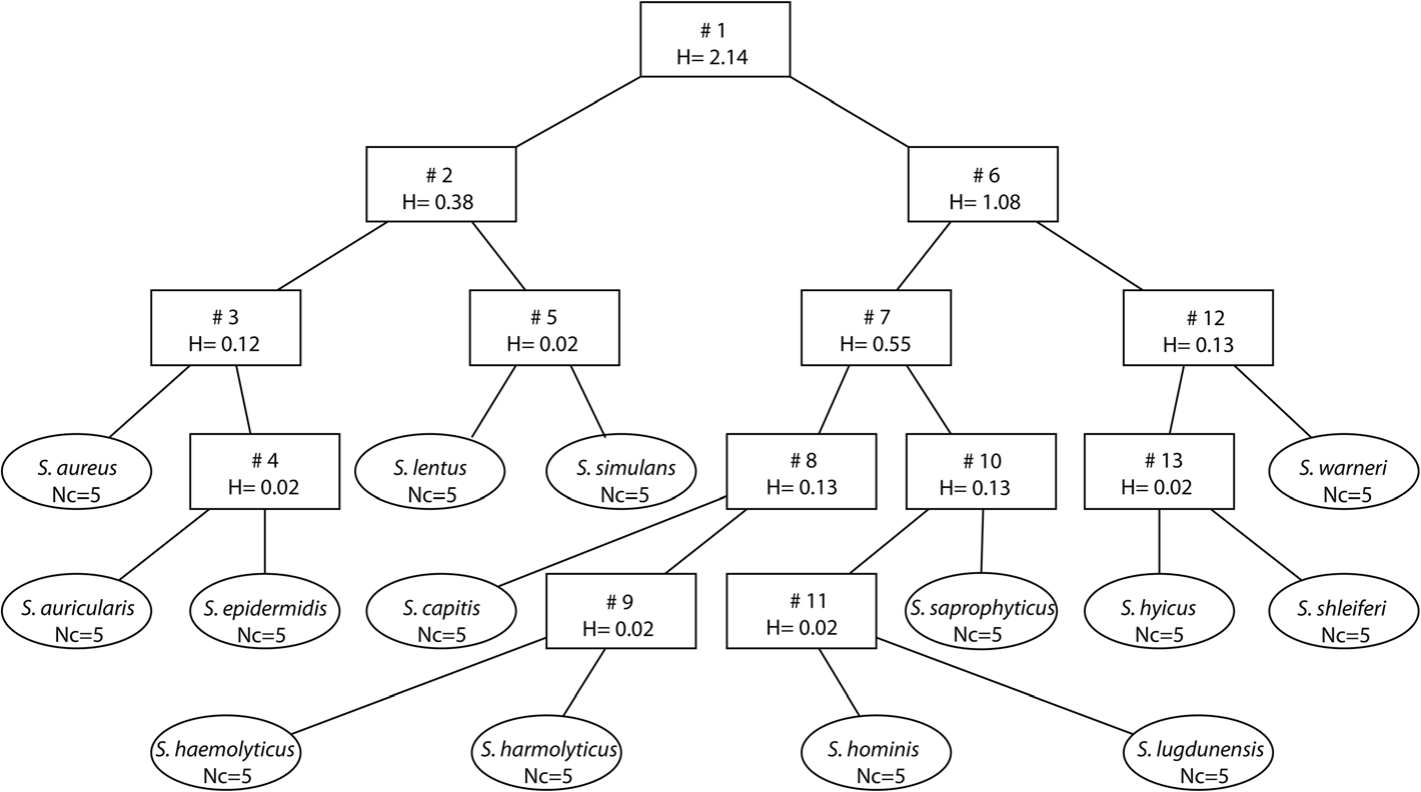
FuRES species-level *Staphylococcus* classification tree. Thirteen rules indicate perfect classification

FuRES analysis (Fig. 4) defined 13 rules indicating perfect classification [29]. Average prediction results for 100 bootstraps were 98.1 ± 0.3 % for FuRES and 90.7 ± 0.3 % for PLS-DA. Bootstrap Latin partition validation randomly divided the data into training and test sets such that the training set contained twice the number of data points when compared to the test set. In addition, validation maintained the same class distributions between training and test sets so that training and test sets would have the same proportion of objects (replicates) from each class (isolate). Three hundred models were built and evaluated for bootstrap analysis. Because each profile was only used once per bootstrap, the results of three Latin partitions were pooled and were comprehensive for all FA profiles. The results from 100 bootstraps were averaged and reported with 95 % confidence intervals. FuRES and PLS-DA, which are much more rigorous than LOSOCV, are a weaker measure with respect to a model’s dependence on training set composition and the accuracy of the data within the prediction set. FuRES consistently outperformed PLS-DA, because it is a nonlinear classifier ideally suited for predicting classes that are binary encoded. PLS-DA, which is designed for calibration of continuous variables, may construct ill-conditioned models (ones with poor predictions) when trying to fit the binary encoded target matrix. This problem often occurs with complex data sets [31].

### Strain-level differentiation

**Fig. 5:**
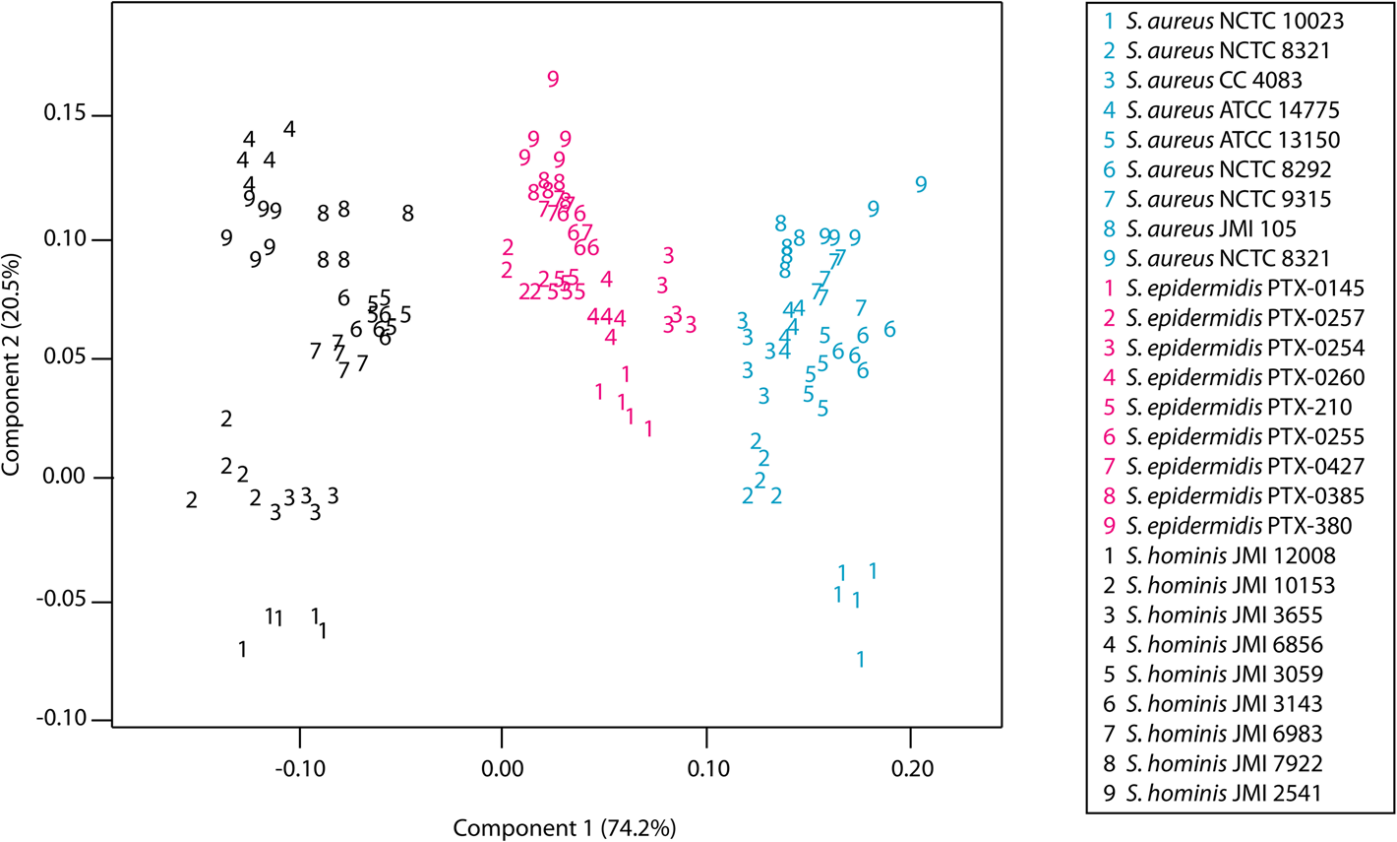
Strain-level PCA differentiation of 18 *Staphylococcus* isolates

**Fig. 6:**
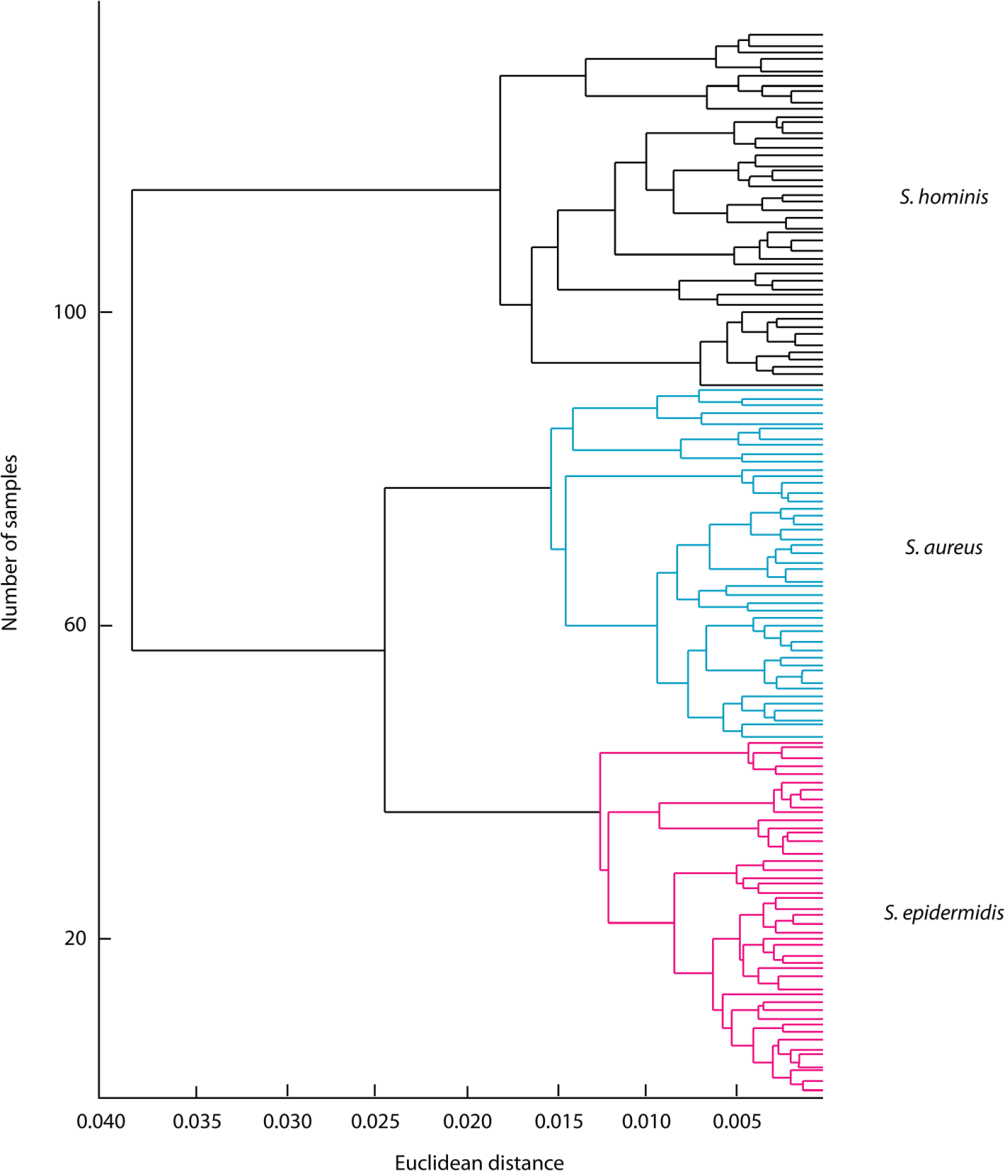
Dendrogramatic representation of *Staphylococcus* strain differentiation. Branch lengths were determined using average linkages and Euclidean distance

The versatility of MOLI MS for strain-level identification was further explored by analyzing extracts of 27 additional strains (Table 1B). Fig. 5 shows a score plot of the first two PCs for this data; a total variance of 94.7 % was defined by the first two PCs. The strains are denoted numerically with each species being represented by a different color. Leave-one-spectrum-out cross-validation of the first ten PC scores correctly identified 100 % (145/145) of the samples at the species level and strain level, showing that all strains plotted independently. Species-level groupings were also seen in the dendrogram in Fig. 6, where each main branch point corresponded to its own individual species.

FuRES and PLS-DA calculations correctly classified the data into 27 strains. These results were obtained with 100 bootstraps and three Latin partitions. FuRES and PLS-DA had 93.9 ± 0.4 % and 84.1 ± 0.4 % prediction rates, respectively. From the PCA scores, it was shown that strains of the same species exhibit profiles that were highly similar.

### MRSA/MSSA differentiation

**Fig. 7:**
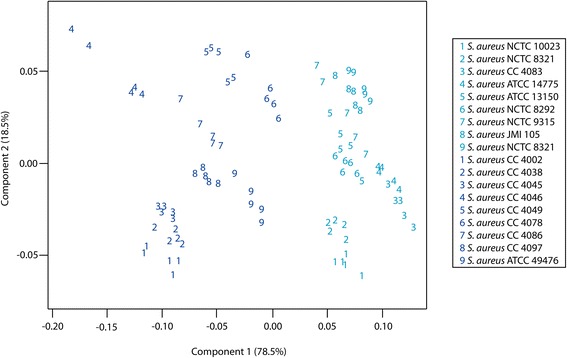
PCA differentiation of MRSA and MSSA. MRSA strains are designated by black and MSSA strains are blue

MALDI protein profiling methods have shown a series of characteristic peaks for identification of *S. aureus* [32]*.* From direct comparison of reference strains, discrimination between MSSA and MRSA was achieved*,* but a uniform signature profile could not be identified to allow for unknown classification [33]. To assess the utility of MOLI MS FA profiling for antibiotic resistance profiling, 18 *S. aureus* strains (nine MRSA and nine MSSA), listed in Table 1C were analyzed. A score plot of the first two components defining 97 % of the total variance is shown in Fig. 7. In this projection, all strains were separated into unique groups according to methicillin resistance/susceptibility. Strain-level classification correctly identified 90/90 total replicates leading to 100 % accuracy using LOSOCV.

**Fig. 8:**
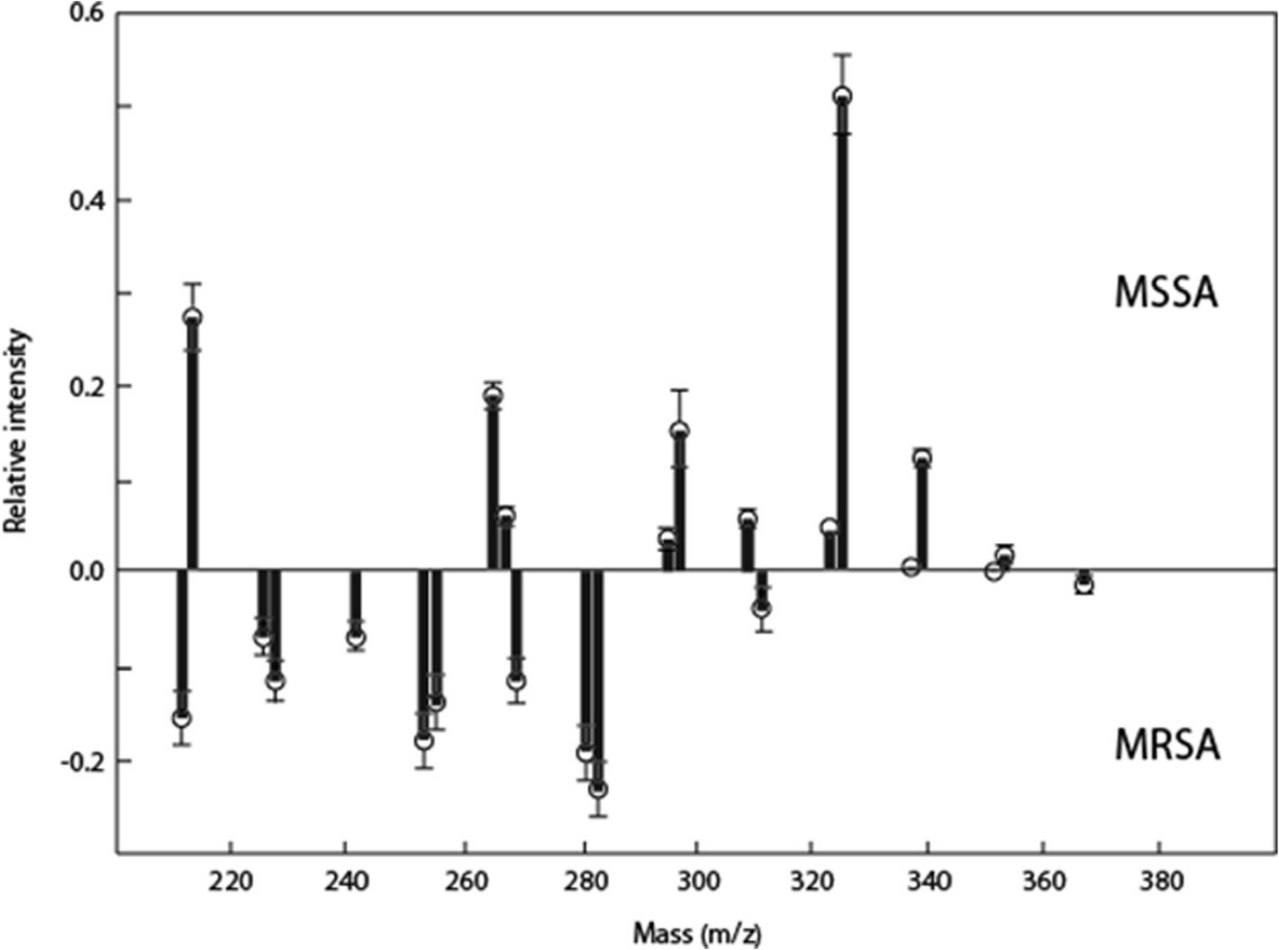
Average of 300 FuRES discriminant weights with 95 % confidence intervals. If the confidence interval intersects the origin that weight is significant, with negative weights corresponding to larger features in MRSA and positive weights to larger features in MSSA

The above data set yielded a FuRES tree with a single rule (figure not shown) indicating perfect separation of the two bacterial classes. Because each of the MRSA and MSSA groups comprised five replicates each of nine different strains, bootstrap Latin partitioning grouped all samples such that no profiles from any given strain were contained in both the training and prediction sets at the same time. The prediction rates for strain-level identification of *S. aureus* were 94.7 ± 0.6 % for FuRES and 93.7 ± 0.5 % for PLS-DA. FuRES discriminant weights, based on a 95 % confidence interval, for MRSA and MSSA classification revealed that odd-numbered fatty acids (C13, C17, C19, C21) were more prevalent in MSSA isolates, while even-numbered fatty acids (C14, C16, C18) were more prevalent in MRSA isolates (Fig. 8). If the confidence interval intersected the origin in the positive or negative direction, that weight was significant. These results were in agreement with other reports in the literature that showed differences in FA composition between daptomycin-resistant *Enterococcus* strains [34].

## Conclusions

We demonstrated the utility of CeO_2_-MOLI MS FA profiling coupled with multivariate statistical analysis for performing strain-level differentiation of various *Staphylococcus* species. The emergence of MRSA and CoNS clinical isolates and the need for rapid clinical intervention has made it increasingly important to differentiate *Staphylococcus* isolates at the species and strain level. LOSOCVs yielded 100 % correct classification at the species and strain level. FuRES classification, with a more rigorous evaluation, also consistently achieved 94 % accuracy. Preliminary analysis differentiating MRSA from MSSA demonstrated the feasibility of simultaneously determining strain identification and antibiotic resistance, which is increasingly important for therapeutic management and infection control. By eliminating the need for secondary testing, this could decrease the delay of drug administration by up to 54 h over conventional diagnostic techniques. Ultimately, as is also the case in protein profiling, construction of a comprehensive database will be necessary for identification of unknown isolates.

## Methods

### Bacterial isolates

Table 1 summarizes the bacteria used in this study. All strains were obtained from an in house collection at CSM, JMI laboratories (North Liberty, IA) and the National Collection of Type Cultures (NCTC) (Salisbury, UK). Bacteria were streaked on brain heart infusion (BHI) medium (BD-Difco, Franklin Lakes, NJ) from cryogenic freezer stocks and cultured at 37 °C for 18 h. as specified in Bruker standard operating procedures for bacterial cultivation.

**Table 1:** *Staphylococcus* species used

Species	Strain
A. Species-level study	
*S. aureus*	ATCC 29213
*S. auricularis*	JMI 66-1339p
*S. capitis*	JMI 186-14645a
*S. epidermidis*	JMI s12410
*S. haemolyticus*	JMI 14138
*S. harmolyticus*	JMI s11298
*S. hominis*	JMI 9382
*S. hyicus*	JMI 15-8308a
*S. lentus*	JMI 7613
*S. lugdunensis*	JMI 112-3379a
*S. saprophyticus*	PTX-0652
*S. shleiferi*	JMI 100-1511a
*S. simulans*	JMI 7295
*S. warneri*	JMI 12019
B. Strain-level study	
*S. aureus*	ATCC 13150
*S. aureus*	ATCC 14775
*S. aureus*	NCTC 9315
*S. aureus*	NCTC 8292
*S. aureus*	NCTC 8321
*S. aureus*	NCTC 10023
*S. aureus*	JMI 105
*S. aureus*	CC 4051
*S. aureus*	CC 4083
*S. epidermidis*	PTX 0254
*S. epidermidis*	PTX 0260
*S. epidermidis*	PTX 0257
*S. epidermidis*	PTX 0255
*S. epidermidis*	PTX 210
*S. epidermidis*	PTX 0145
*S. epidermidis*	PTX 0385
*S. epidermidis*	PTX 0427
*S. epidermidis*	PTX 380
*S. hominis*	JMI 12008
*S. hominis*	JMI 2541
*S. hominis*	JMI 7922
*S. hominis*	JMI 10153
*S. hominis*	JMI 6983
*S. hominis*	JMI 6856
*S. hominis*	JMI 3655
*S. hominis*	JMI 3059
*S. hominis*	JMI 3143
C. Resistance study	
*S. aureus*	ATCC 13150
*S. aureus*	ATCC 14775
*S. aureus*	NCTC 9315
*S. aureus*	NCTC 8292
*S. aureus*	NCTC 8321
*S. aureus*	NCTC 10023
*S. aureus*	JMI 105
*S. aureus*	CC 4051
*S. aureus*	CC 4083
*S. aureus*	ATCC 49476^a^
*S. aureus*	CC 4002^a^
*S. aureus*	CC 4038^a^
*S. aureus*	CC 4045^a^
*S. aureus*	CC 4048^a^
*S. aureus*	CC 4049^a^
*S. aureus*	CC 4078^a^
*S. aureus*	CC 4086^a^
*S. aureus*	CC 4097^a^

~ MRSA strains are denoted by ^a^

**Table 2:** Fatty acids used in Principal Component Analysis

Fatty acid	[M-H]^−^
C13:1	211
C13:0	213
C14:1	225
C14:0	227
C15:0	241
C16:1	253
C16:0	255
C17:2	265
C17:1	267
C17:0	269
C18:2	279
C18:1	281
C18:0	283
C19:1	295
C19:0	297
C20:1	309
C20:0	311
C21:1	323
C21:0	325
C22:1	337
C22:0	339
C23:1	351
C23:0	353
C24:1	365
C24:0	367
C25:1	379
C25:0	381
C26:1	393
C26:0	395

### Lipid extraction

Lipids were extracted as previously described [24, 27]. Briefly, individual colonies were suspended in 50 μL of a 1:2 v/v methanol/chloroform (Pharmco-AAPER, Shelbyville KY and Fischer, Pittsburgh PA, respectively) and vortexed for 120 s. to allow for cell disruption. An equal volume of phosphate buffer saline (PBS) at a pH of 7.4 was added prior to additional vortexing to facilitate phase separation. Extracts were centrifuged prior to MALDI sample preparation.

### Mass spectrometry

Sample preparation for MOLI MS analysis was carried out as previously described [25]. Briefly, 100 mg of CeO_2_ (Cermac Inc., Milwaukee, WI) was suspended in 1 mL of *n*-hexane (Sigma Aldrich) prior to spotting 1 μL of the resulting slurry on a standard Bruker stainless steel MALDI plate. Two μL of each lipid extract was deposited directly on a CeO_2_ spot and allowed to air dry prior to analysis. MOLI-MS measurements were performed with a Bruker Ultraflextreme MALDI-TOF MS (Bruker Daltronics, Billerica, MA) in negative-ion reflectron mode with a grid voltage of 50.3 %, a delayed extraction time of 120 ns, and a sampling frequency of 1 kHz on a 355 nm Nd:YAG laser. Five replicates of each isolate were analyzed as 500 shot composites using automated laser rastering to ensure instrument stability.

### Data analysis

Mass spectra were exported as ASCII files and processed using a Python algorithm to select and centroid 29 specific fatty acid peaks (Table 2), and scale each peak to total ion intensity. Processed data were written as.xls files for import into R (Ver. 3.0.2, R Foundation, Vienna, Austria) as a data frame. The prcomp()function mean centered and calculated PCA scores before plotting with the built-in plot()function. Leave-one-spectrum-out cross-validation (LOSOCV) was performed using linear discriminant analysis to validate the classification rate.

Processed fatty acid profiles were further analyzed with MATLAB 2014a (Mathworks, Natick, MA). Generalized prediction rates were measured using three Latin partitions and 100 bootstraps [29]. Two classifiers were evaluated: a fuzzy rule-building expert system (FuRES) [29] and partial least squares discriminant analysis (PLS-DA) [30]. The PLS-DA algorithm used two Latin partitions and ten bootstraps to calculate average pooled prediction errors [31]. The number of components (i.e., latent variables) that minimized error was selected and used to build a model from the set of training data, which was then used as a prediction set. Training data consisted of a set of profiles used to build the classifiers; the test data was the set of profiles used to evaluate the performance of these classifiers. Hierarchical cluster analysis was used to generate dendrograms and graphically illustrate linkage distances (Euclidean distances) obtained from an agglomerative algorithm. The distances were between pairs of profiles or between the averages of profiles from subclusters.

## References

[1] Lowy FD (1998). *Staphylococcus aureus* infections. N Engl J Med.

[2] Centres for Disease Control and Prevention (US). Antibiotic resistance threats in the United States, 2013. Centres for Disease Control and Prevention, US Department of Health and Human Services; 2013.

[3] Centers for Disease Control and Prevention. Active Bacterial Core Surveillance Report, Emerging Infections Program Network, Methicillin-Resistant Staphylococcus aureus; 2011.

[4] Kumar A (2006). Duration of hypotension before initiation of effective antimicrobial therapy is the critical determinant of survival in human septic shock*. Crit Care Med.

[5] Flayhart D (2005). Multicenter evaluation of BBL CHROMagar MRSA medium for direct detection of methicillin-resistant *Staphylococcus aureus* from surveillance cultures of the anterior nares. J Clin Microbiol.

[6] Kloos WE, Schleifer KH (1975). Simplified scheme for routine identification of human *Staphylococcus* species. J Clin Microbiol.

[7] Kloos WE, Wolfshohl JF (1982). Identification of *Staphylococcus* species with the API STAPH-IDENT system. J Clin Microbiol.

[8] Hussain ZAFAR (1986). Comparison of the MicroScan system with the API Staph-Ident system for species identification of coagulase-negative staphylococci. J Clin Microbiol.

[9] Sasaki T (2007). Reclassification of phenotypically identified *Staphylococcus intermedius* strains. J Clin Microbiol.

[10] Pottumarthy S (2004). Clinical isolates of *Staphylococcus intermedius* masquerading as methicillin-resistant *Staphylococcus* aureus. J Clin Microbiol.

[11] Brakstad OG, Maeland JA, Tveten Y (1993). Multiplex polymerase chain reaction for detection of genes for *Staphylococcus aureus* thermonuclease and methicillin resistance and correlation with oxacillin resistance. Apmis.

[12] Martineau F (2001). Development of a PCR assay for identification of staphylococci at genus and species levels. J Clin Microbiol.

[13] Bukau B, Horwich AL (1998). The Hsp70 and Hsp60 chaperone machines. Cell.

[14] Edwards U, Rogall T, Blöcker H, Emde M, Böttger EC (1989). Isolation and direct complete nucleotide determination of entire genes. Characterization of a gene coding for 16S ribosomal RNA. Nucleic Acids Res.

[15] Bhowmick T (2013). Controlled multicenter evaluation of a bacteriophage-based method for rapid detection of *Staphylococcus aureus* in positive blood cultures. J Clin Microbiol.

[16] Smith D. Accurate Detection of Nasal MRSA Carriage by the Bacteriophage Amplification Test. 46th Annual Meeting. Idsa; 2008.

[17] David MZ, Daum RS (2010). Community-associated methicillin-resistant *Staphylococcus aureus*: epidemiology and clinical consequences of an emerging epidemic. Clin Microbiol Rev.

[18] Sievert DM (2008). Vancomycin-resistant *Staphylococcus aureus* in the United States, 2002–2006. Clin Infect Dis.

[19] Thati V, Shivannavar CT, Gaddad SM (2011). Vancomycin resistance among methicillin resistant *Staphylococcus aureus* isolates from intensive care units of tertiary care hospitals in Hyderabad. Indian J Med Res.

[20] Rogers KL, Fey PD, Rupp ME (2009). Coagulase-negative staphylococcal infections. Infect Dis Clin N Am.

[21] Harris LG, El-Bouri K, Johnston S (2010). Rapid identification of staphylococci from prosthetic joint infections using MALDI-TOF mass-spectrometry. Int J Artif Organs.

[22] Dubois D (2010). Identification of a variety of *Staphylococcus* species by matrix-assisted laser desorption ionization-time of flight mass spectrometry. J Clin Microbiol.

[23] Rajakaruna L (2009). High throughput identification of clinical isolates of S*taphylococcus aureus* using MALDI-TOF-MS of intact cells. Infect Genet Evol.

[24] Voorhees KJ (2013). Modified MALDI MS fatty acid profiling for bacterial identification. J Mass Spectrom.

[25] McAlpin CR, Voorhees KJ, Corpuz AR, Richards RM (2012). Analysis of lipids: metal oxide laser ionization mass spectrometry. Anal Chem.

[26] Voorhees KJ, McAlpin CR, Cox CR (2012). Lipid profiling using catalytic pyrolysis/metal oxide laser ionization-mass spectrometry. J Anal Appl Pyrolysis.

[27] Voorhees KJ, Saichek NR, Jensen KR, Harrington PB, Cox CR (2014). Comparison of metal oxide catalysts for pyrolytic MOLI–MS bacterial identification. J Anal Appl Pyrolysis.

[28] Cox CR, Jensen KR, Saichek NR, Voorhees KJ (2015). Strain-level bacterial identification by CeO_2_-catalyzed MALDI-TOF MS fatty acid analysis and comparison to commercial protein-based methods. Nat Sci Rep.

[29] Harrington PB (1991). Fuzzy multivariate rule‐building expert systems: minimal neural networks. J Chemom.

[30] Harrington PB, Kister J, Artaud J, Dupuy N (2009). Automated principal component-based orthogonal signal correction applied to fused near infrared− mid-infrared spectra of French olive oils. Anal Chem.

[31] Harrington PB (2006). Statistical validation of classification and calibration models using bootstrapped Latin partitions. TrAC Trends Anal Chem.

[32] Edwards-Jones V (2000). Rapid discrimination between methicillin-sensitive and methicillin-resistant *Staphylococcus aureus* by intact cell mass spectrometry. J Med Microbiol.

[33] Bernardo K (2002). Identification and discrimination of *Staphylococcus aureus* strains using matrix‐assisted laser desorption/ionization‐time of flight mass spectrometry. Proteomics.

[34] Mishra NN (2012). Daptomycin resistance in enterococci is associated with distinct alterations of cell membrane phospholipid content. PLoS One.

